# Roles of fMRI and Wada tests in the presurgical evaluation of language functions in temporal lobe epilepsy

**DOI:** 10.3389/fneur.2022.884730

**Published:** 2022-09-30

**Authors:** Andreu Massot-Tarrús, Seyed M. Mirsattari

**Affiliations:** ^1^Department of Neurology, F. Ass. Mútua Terrassa, Terrassa, Spain; ^2^Department of Clinical Neurological Sciences, Western University, London, ON, Canada; ^3^Department of Medical Biophysics, Western University, London, ON, Canada; ^4^Department of Medical Imaging, Western University, London, ON, Canada; ^5^Department of Psychology, Western University, London, ON, Canada

**Keywords:** intracarotid amobarbital test, functional MRI, resting-state, epilepsy surgery, naming, outcome

## Abstract

Surgical treatment of pharmacoresistant temporal lobe epilepsy (TLE) carries risks for language function that can significantly affect the quality of life. Predicting the risks of decline in language functions before surgery is, consequently, just as important as predicting the chances of becoming seizure-free. The intracarotid amobarbital test, generally known as the Wada test (WT), has been traditionally used to determine language lateralization and to estimate their potential decline after surgery. However, the test is invasive and it does not localize the language functions. Therefore, other noninvasive methods have been proposed, of which functional magnetic resonance (fMRI) has the greatest potential. Functional MRI allows localization of language areas. It has good concordance with the WT for language lateralization, and it is of predictive value for postsurgical naming outcomes. Consequently, fMRI has progressively replaced WT for presurgical language evaluation. The objective of this manuscript is to review the most relevant aspects of language functions in TLE and the current role of fMRI and WT in the presurgical evaluation of language. First, we will provide context by revising the language network distribution and the effects of TLE on them. Then, we will assess the functional outcomes following various forms of TLE surgery and measures to reduce postoperative language decline. Finally, we will discuss the current indications for WT and fMRI and the potential usefulness of the resting-state fMRI technique.

## Introduction

Surgery for temporal lobe epilepsy (TLE) accounts for 60% to 80% of all surgeries for pharmacoresistant epilepsies. It allows sustained seizure freedom to be achieved in ~66% of the cases after more than 5 years (1). However, it may entail risks in terms of language deficits that can be more detrimental to the patient's quality of life than the seizures themselves (2). Therefore, it is important to predict the risks of language deficits before surgery along with the odds of becoming seizure-free afterward.

The intracarotid amobarbital test, generally known as the Wada test (WT), has traditionally been used to determine language lateralization to estimate the risks of decline after surgery. It is, however, an invasive test that does not localize language functions. Therefore, multiple noninvasive methods have been devised to replace WT. Among these, functional magnetic resonance (fMRI) has the greatest potential (3, 4), as it permits localization and lateralization of all language regions. Functional MRI has a good concordance with the WT for language lateralization (5), and it has a good predictive value for naming outcomes after surgery (6, 7). Therefore, fMRI has replaced the WT for routine presurgical evaluation of language functions in most comprehensive epilepsy centers (8).

The objective of the present manuscript is to review the most relevant aspects of language functions in TLE and the role of fMRI and WT in the presurgical evaluation of language. We will first provide context by reviewing the distribution of the language networks and the effect of TLE and epilepsy surgery on them. Later on, we will discuss the principles of the WT and fMRI techniques and compare their usefulness to predict postsurgical language outcomes. Finally, we will discuss the current role of the WT and fMRI in the presurgical assessment of language, the surgical measures to prevent language decline, and the potential role of the resting-state fMRI (rs-fMRI) technique.

## Language

### How are the neural networks for language distributed?

At the end of the nineteenth century, Broca and Wernicke recognized that lesions in the left triangular and opercular parts of the inferior frontal gyrus (“Broca's area”) caused poorly articulated speech and lesions to the posterior part of the left middle and superior temporal gyrus, angular and supramarginal gyrus (“Wernicke's area”) caused meaningless speech (9). Lichtheim and Geschwind (10) subsequently added the elements of conduction aphasia and cortical disconnection, showing that damage to the fibers that connect different cortical areas in the inferior parietal lobe can result in language impairment (10, 11).

Knowledge regarding the language areas and their interactions has evolved substantially in recent decades as a result of the development of neuroimaging techniques and electrocortical stimulation mapping (ESM) (9). These techniques have revealed new areas in both hemispheres activated during languages (12, 13), such as the inferior and anterior temporal cortex (14) and a wide range of frontal regions (11). The anatomic location and functions of Broca's (also known as anterior language area) and Wernicke's (or posterior language area) have also been called into question. The classic Broca's area can extend to the pars orbitalis of the frontal operculum (15), and it appears to be composed of two subregions, one specific for speech production and another engaged in multiple cognitive tasks such as arithmetic and spatial and verbal working memory (11). Language comprehension in turn is supported by a widely distributed bilateral temporal, parietal, and frontal network in which the classic Wernicke's area is mainly involved in the mental representations of phoneme sequences and short-term memory tasks (16).

**Figure 1:**
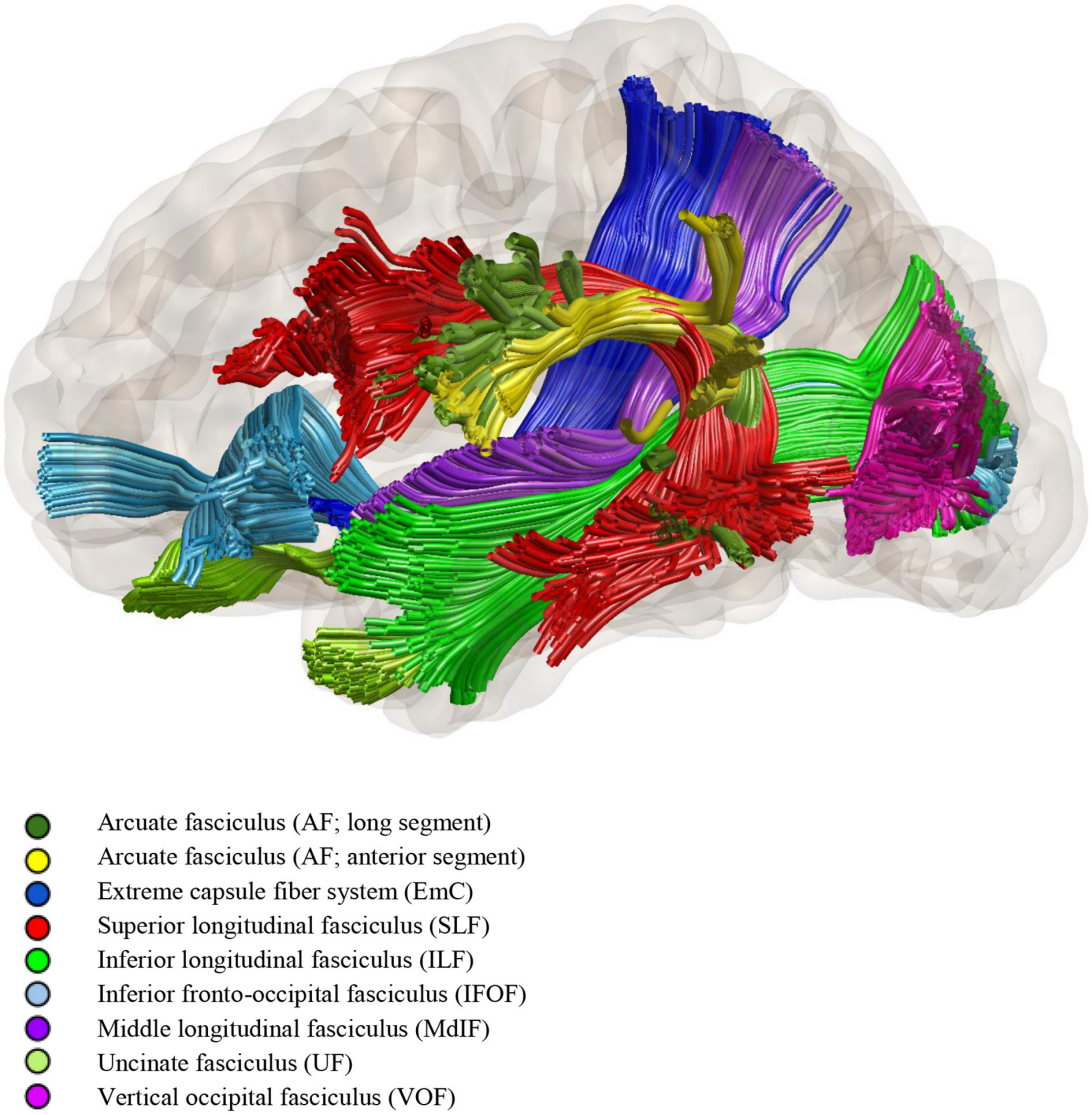
Diffusion tensor tractography of the fascicles involved in language function (by Dr. Loxlan W. Kasa).

Modern network-based models are composed of interconnected streams, involving both cortical and subcortical areas. Hickok and Poeppel proposed a “dual-stream model” based on dorsal (motor) and ventral (semantic) white matter streams traveling dorsally and ventrally to the Sylvian fissure. The dorsal stream is strongly lateralized to the left cerebral hemisphere, goes from the posterior superior temporal lobe (TL) to the inferior frontal gyrus and premotor cortex, and involves the arcuate and superior longitudinal fasciculi. The ventral stream in turn connects the superior temporal gyrus to the pars orbitalis and triangularis of the inferior frontal gyrus, and includes the middle and inferior longitudinal fasciculi, uncinate and inferior fronto-occipital fasciculi (11). For a schematic representation of the fascicles involved in language function see Figure 1.

The dorsal pathway is linked to the sensory-motor mapping of sound for articulation and auditory feedback control. It is, therefore, important in the repetition and combination of speech elements (phonemes, words) into a sequence. The ventral stream, on the other hand, is responsible for mapping the auditory input onto conceptual and semantic representations (17). It is mostly underpinned by the inferior fronto-occipital fasciculus, which subserves language semantics. The inferior longitudinal fasciculus, on the other hand, is involved in lexical retrieval and reading, and the uncinate fasciculus controls retrieval and selection of semantic knowledge during word comprehension (18).

At a cortical level, the functions of the TL have been divided as follows: (1) The superior temporal gyrus is associated with phonological processing. Specifically, the acoustic processing of linguistic stimuli is mediated by the auditory cortex in the midportion of the superior temporal gyrus in both hemispheres, with the left hemisphere specializing in acoustic phonology (19). (2) The middle temporal gyrus is considered an integration hub for semantics and phonology needed for sentence comprehension (20, 21). (3) The inferior temporal gyrus, angular gyrus, and anterior temporal pole are involved in semantic storage and grammatical sentence discrimination (22–27). (4) The fusiform gyrus is associated with visual language and verbal word discrimination (28). Other functions identified in the basal temporal area include auditory and visual naming, as well as verbal comprehension (29). Essentially, auditory naming is localized to the anterior TL cortex, while visual naming primarily relies on the mid-posterior temporal regions (30). However, there is an important interindividual variability in the cortical temporal regions involved in each language function, due to the higher degree of plasticity at the cortical level.

At a cortical level, the eloquent language cortex can be divided into indispensable eloquent cortex (whose resection would be associated with permanent and significant deficit) and dispensable eloquent cortex (whose resection would lead mostly to minimal and reversible functional deficit) (15). Based on ESM findings, discrete and limited patches of indispensable language eloquent cortex had been located in the posterior part of the inferior frontal gyrus (pars opercularis), posterior part of the superior temporal gyrus, and angular gyrus (15, 31). In contrast, the basal temporal language area (composed of the fusiform, inferior temporal and parahippocampal gyri, and lateral occipitotemporal sulcus) is considered a dispensable eloquent cortex (15). Other dispensable eloquent cortexes would be the anterior part of the superior temporal gyrus (32, 33), the anterior temporal pole (34), middle temporal gyrus (29, 35), and the hippocampus—which have also been involved in the multi-modal naming network by some authors (15).

### The effects of TLE on language function

Epileptic activity disturbs the language networks and promotes brain plasticity (36, 37). Atypical, that is, bilateral or right-hemispheric language lateralization, is more frequently seen in patients with left compared to right TLE (33–41 vs. 22%) or healthy individuals (4–27%). This is particularly so if they are left-handed (8, 38), the epilepsy is caused by a structural etiology (39), started before 5 years of age (40), or seizures are frequent (38, 41). Moreover, TLE can affect the reorganization of temporal and frontal language areas differently (42). There are, consequently, as many categorical patterns of language lateralization as combinations of anterior and posterior language regions in the left, right, or bilateral hemisphere (43).

There can also be a reorganization of language functions within the same hemisphere due to epileptic foci, often to an adjacent perisylvian region (44). This typically occurs in patients with space-occupying lesions, although it has also been observed in non-lesional TLE (34). For example, patients with left TLE had reduced activation during a verbal fluency task in the left superior temporal gyrus in fMRI, but increased activation in the inferior, middle temporal, and fusiform gyri (45). Additionally, a longer duration of left TLE causes weaker activation of the anterior middle temporal gyrus and fusiform gyrus during auditory and picture naming tasks (46), and poorer functional connectivity of the left temporal pole (47). Posterior displacement of visual and auditory naming areas or reorganization of the tracts connecting language brain areas within the left TL has also been reported in individuals with hippocampal sclerosis (41). Such neuroplasticity implies that surgery can be performed in brain areas previously considered inoperable such as the Wernicke's area involved in the epileptic zone (middle and posterior temporal cortex corresponding to scalp EEG electrodes T3-T5-A1) in left neocortical TLE (18). The high variability of cortical eloquent areas for language highlights the need for individualized approaches to each patient and brain region.

About 39% of patients with long-lasting left TLE suffer from language deficits (48). These usually consist of verb generation fluency, visual and auditory naming, and word-finding difficulties (41). Patients with non-dominant TLE also perform worse in language function than healthy controls and patients with extratemporal lobe epilepsy (48). The mechanism underlying naming impairment in TLE involves lexical retrieval. Thus, the patients can recognize and describe the objects or faces presented but have difficulties retrieving their specific names, producing the typical “tip of the tongue” phenomenon with phonological paraphasia (34, 49, 50). This suggests that there is a one-way disruption in the semantic-lexical pathway. That is to say, information can still flow from lexical to semantic but not the other way around. It may involve the ventral visual pathway and possibly the inferior segment of the arcuate fasciculus, as well as phonological processing areas such as the left superior temporal gyrus and supramarginal gyrus (41).

In contrast, non-dominant TL dysfunction leads to recognition deficits. These patients have difficulty recognizing famous faces and landmarks. This dysfunction is thought to result from damage to the ventral visual processing stream (34). Deficits in category fluency are present in both dominant and non-dominant TLE, as well as in frontal lobe epilepsy (FLE), since this task requires the executive function mediated by the frontal lobe and a semantic memory mediated by the TL (34).

### Functional outcome of language following the various modalities of TLE surgery

Left ATL causes some degree of worsening in naming and reading in 34% to 40% of patients. In contrast, right ATL causes a decline of these functions in only 5% of cases and even some improvement in 4–32% (30, 34, 51). Semantic fluency (e.g., generating animal names) declines following dominant, and perhaps non-dominant, TL resections (34, 52). By contrast, letter fluency tasks (e.g., generating words that start with a specific letter) typically do not decline or can even improve after successful left TL surgery (34, 53). The naming decline following a dominant TL resection is typically characterized by a lexical retrieval deficit (i.e., inability to recall names in a variety of contexts with preserved semantic knowledge) (34), particularly for human faces and animals (44). Non-dominant ATL frequently leads to deficits in the recognition of people, animals, or landmarks (34).

Anomia is the most common cognitive deficit after the resection of the dominant basal temporal language area but it usually recovers, at least partially, after a few months (15). There is typically a greater impairment in visual compared to auditory naming attributed to the posterior displacement of the auditory naming network in TLE (30) and the resection of the visual processing stream and the fusiform gyrus (34). Knowing the hemispheric dominance for language, it is, therefore, necessary to provide patients with an accurate estimation of the language deficits they may suffer after TLE surgery in order to give informed consent. Language function also needs to be evaluated presurgically since a low performance implies that language structures are already impaired and consequently a lower risk of a significant decline in case of surgery in the dominant hemisphere (54).

Reversal of atypical hemispheric language lateralization has been observed after successful epilepsy surgery in both frontal perisylvian and temporal language regions. This indicates that atypical dominance due to epileptic activity may be a temporary adaptative response (44). Displacement of language regions has also been observed following surgical excision of gliomas infiltrating the posterior part of the left superior temporal gyrus (classic Wernicke's area). It involved the recruitment of not only the immediately adjacent structures (i.e., the supramarginal gyrus) but also remote sites within the same hemisphere (i.e., the pars triangularis of the inferior frontal gyrus) and contralateral sites thanks to transcallosal disinhibition (18, 55). Activation of regions not specific for language (i.e., the anterior cingulate cortex or the mesial frontal gyrus) during verb generation tasks has also been observed after right and left ATL, indicating an increased reliance on a more cognitive control and response selection (44, 56). Interestingly, Bonelli et al. (57) found that patients with left TLE without postoperative naming decline recruited the residual left posterior hippocampus for word retrieval, while those with language decline relied on contralateral frontal regions (57). These findings may indicate that interhemispheric reorganization of naming to contralateral frontal regions is less effective than the utilization of the remaining posterior hippocampus. Thus, the circumstances under which intra- vs. interhemispheric reorganization of language regions occur after TLE surgery and the relative benefit of each of these patterns are still unclear, but the preservation of the posterior hippocampus may facilitate naming maintenance.

## Wada test

The WT simulates the effects of brain surgery through the temporary inactivation of one hemisphere. This involves the use of short-acting barbiturates such as sodium amobarbital or methohexital, or ultra-short-acting non-barbiturates anesthetics such as etomidate (58–60). Intracarotid injection of sodium amobarbital was originally used by Juhn Wada in 1949 to treat a case of status epilepticus. The resulting transient aphasia in the patient led to the consideration that the procedure could be used to determine the hemispheric lateralization for language (61).

### Mechanism of WT

First, a cerebral arteriogram is conducted to rule out abnormalities that can predispose to cross-flow between the two cerebral hemispheres or to the basilar artery implying a risk of respiratory and consciousness impairments following injection of sodium amobarbital. Then, a bolus of 75–125 mg (depending on the weight of the patient) of sodium amobarbital is injected over 5–10 s into the internal carotid artery with a catheter placed *via* transfemoral approach, followed by incremental doses of 12.5 mg, as required, to produce contralateral hemiplegia and development of a theta-delta EEG pattern in the studied hemisphere. Alternatively, the procedure can be made using methohexital (3 mg bolus over 3–5 s, followed by additional doses of 2 mg if required) (60) or etomidate (2 mg bolus, followed by a continuous infusion of 0.003–0.004 mg/kg/minute at a rate of 6 ml/h. The infusions may be switched to a bolus-type procedure, if major patient reactions such as reduced cooperation or obtundation warrant reduction of drug effect) (62).

The procedure can be repeated on the contralateral side after a minimum of 30 min. Language testing begins immediately after the contralateral arm paresis and EEG changes, and it continues depending on the patient's clinical response and drug effects. The side ipsilateral to the epilepsy focus is tested first in case the test needs to be terminated prematurely (5, 63). Simultaneous continuous EEG recordings allow the exclusion of epileptic seizures that could interfere with the evaluation.

### WT for assessing language

Counting and naming the months of the year or different objects are commonly used to evaluate expressive language functions. On the other hand, responses to verbal commands, repetition, and reading are used to assess receptive language. The procedure usually consists of (1) testing for comprehension of one- and two-step commands (the most complex being commands involving inverted syntax); (2) naming of objects or parts of objects presented visually; (3) reading of sentences; and (4) repetition of simple phrases. The performance on each of these tests is scored as either normal or mildly, moderately, or severely deficient (19).

Language dominance is classified as left, right, or bilateral, depending on the degree of impairment observed during the right and left hemisphere injections. A laterality index (LI), consisting of the percentage of correct answers during the right minus the left injections, can be calculated. It usually ranges from +100 to −100, with an arbitrary cut-off commonly of ±50 producing the following categories: left (LI ≥ +50), right (LI ≤ −50), and bilateral (LI between −50 and +50) language dominance (64).

### Limitations of the WT

A serious complication occurs in 1.1–11% of IAT procedures. These include stroke in 0.6% to 1.2% of cases, carotid dissection in 0.7% (especially in older patients), bleeding from the catheter insertion site in 0.6%, and allergic reaction to the contrast agent in 0.3% (65, 66).

The WT is not feasible in patients with an altered vasculature that may cause cross-flow between hemispheres or to the basilar artery (8, 67). Nor can it be used in patients who are allergic to iodine-based contrast. Moreover, some antiseizure medications such as carbonic anhydrase inhibitors (e.g., acetazolamide, topiramate, and zonisamide) can inhibit the effects of amobarbital (68). Furthermore, its validity relies on good cooperation by the patient. It is, therefore, difficult to complete reliably in children or mentally impaired individuals. Finally, an optimal standardized protocol or clear definition of test failure has not been established, and those used differ broadly across epilepsy centers, thus limiting the comparison of the results (69).

### Functional magnetic resonance imaging and its mechanism

Functional MRI is a non-invasive technique based on blood oxygen level-dependent (BOLD) contrast created due to a difference in the oxyhemoglobin and deoxyhemoglobin levels in the activated areas of the brain. Compared to the WT, this technique is much more cost-effective, safer, and produces sedation-free results.

The most commonly used designs for fMRI studies are block designs. These involve presenting a condition continuously for an extended time interval (block) to maintain cognitive engagement altered by rest blocks, irrespective of performance. An event-related design, on the other hand, consists of distinct and short-duration events that are time-locked and can be presented randomly. The advantages of employing a blocked design include its robustness as well as increased statistical power and sensitivity. It consequently has a good ability to differentiate between different conditions (70). Event-related designs, on the other hand, allow the separation of trials based on the participant's performance (i.e., remembered vs. forgotten items). They are, therefore, more focused on brain processes of clinical interest, but they may not result in strong activation due to reduced statistical power. Event-related designs show reliable activation of language areas (71). However, block-design paradigms are used more commonly in clinical settings to increase the sensitivity of the test.

### Functional MRI paradigms for language lateralization

Different stimulation paradigms are used for language lateralization. These comprise language production tasks—such as object naming, verb generation, and so on—and language comprehension tasks—such as sentence comprehension, rhyme detection, and so on. Some studies employ passive listening to speech, whereas others require participants to respond to the speech sounds according to a particular criterion. Active tasks focus the participant's attention on a specific aspect of a stimulus, such as its form or meaning. This is assumed to cause “top-down” activation of the neural systems relevant for processing the attended information (72).

With verb-generation tasks, the participants are instructed to generate action verbs for given nouns (e.g., for the word “apple” one might think of “to cut” or “to eat”). The control task involves the presentation of nonsense collections of letters. These language production tasks provide consistent activation within the frontal language areas.

With rhyme detection tasks, the participants are asked to determine whether two visually presented common English words rhyme, such as dial-file, and then press the corresponding button on a response pad. The control task may involve participants looking at a cross sign. These language comprehension tasks activate additional brain networks, including the anterior and posterior language areas and areas involved in memory functions (51, 73).

To activate the more anterior temporal region, Hamberger et al. developed an auditory naming task that requires the participants to name nouns in response to an auditory definition (e.g., “The person who flies a plane?”) to activate auditory naming sites (71).

### Region of interest analysis and laterality index in fMRI testing for language functions

When analyzing the fMRI data for language testing, the four regions of interest (ROI) most commonly focused on are the frontotemporal (Broca's area), temporoparietal (Wernicke's area), both Broca's and Wernicke's areas, and the whole hemisphere. Using the whole hemisphere or the combination of ROIs in each hemisphere is more sensitive for language lateralization. It has also been reported that, compared to the temporal-parietal ROI, the frontal lobe ROI appears to be more sensitive and slightly more specific for language lateralization (51). However, the temporal-parietal ROI has more direct clinical relevance for preoperative evaluation. This is especially so since patients with TLE may occasionally present with dissociation between frontal and temporal dominance (51).

The laterality index (LI) is utilized to measure hemispheric dominance for language functions. The LI can be calculated using the formula LI = [(VL – VR) / (VL + VR)] × 100, where VL and VR are the activation volumes or the number of active voxels for the left and right hemispheres, respectively (71). This calculation yields a value ranging from +1 (or +100) for patients with pure left hemispheric lateralization to −1 (or −100) for patients with pure right-hemispheric lateralization. An arbitrary threshold of ± 0.2 (or ± 20) is generally used, with values above +0.2 (or +0.2) considered to be indicative of typical left-hemispheric dominance for language, values below −0.2 (or −20) denoting right hemispheric dominance, and intermediate values indicative of bilateral or mixed dominance. However, some might argue that only an LI of 0 reflects true mixed dominance, whereas others consider laterality to be better reflected by an LI >0.75 (8). Some authors avoid an arbitrary LI cut-off point and instead use visual analysis (8). This subjective analysis appears to be less sensitive and specific, however, than quantitative methods (51).

### Limitations of fMRI studies for assessing language

Functional MRI, though non-invasive, cannot be performed in patients with a cardiac pacemaker, other implanted ferromagnetic materials, or who are claustrophobic. The patients have to lie motionless and perform the tasks with the utmost cooperation. This makes fMRI less suitable for younger children and cognitively low-functioning patients. There are, however, simplified protocols that have been adapted to overcome this limitation (74). Further problems with fMRI include the lack of a consensus on the optimal fMRI protocol for language testing (75). The results also depend on the data analysis software and image pre-processing pipeline, as well as the threshold and ROI chosen. Functional MRI also fails to differentiate between areas activated by the task or by epileptic activity, and no activation is evident in the immediate postictal period. Other factors, such as large structural lesions and cerebral vascular malformations, may also disrupt fMRI activations (76, 77). Moreover, fMRI results are likely to be compromised by the effects of anti-seizure medications taken by the patients (54, 78).

### Reliability and prognostic value of the Wada test for language after TLE surgery

The prognostic value of the WT in TLE surgery is based on the assumption that the risk for language decline after ATL is greater when language lateralizes toward the hemisphere to be operated on. However, the WT has been proven to be reliable and consistent when it identifies left-hemispheric dominance but not when it identifies right-hemispheric or bilateral representation of language function (79–81). Actually, only one study specifically examined the predictive value of the WT in individuals with right-hemispheric dominance (82). This study concluded that the WT provided some prognostic information on naming outcomes after left ATL, albeit with lower specificity and sensitivity than fMRI. With regard to patients with bilateral language representation in the WT, the data are contradictory: although the risk for language decline after left ATL would conceivably be lower in this subgroup of patients, Janecek et al. reported a similar greater decline in naming function after left (50% decline) vs. right (only 10%) ATL in individuals with a bilateral representation of language in the WT (64). Kovac et al. described an even higher decline in naming after surgery in such patients compared to those with left-hemispheric dominance (83).

Therefore, left dominance by WT is strong evidence for lateralization of language function in the left hemisphere and those patients would not require further studies before extensive right temporal lobectomy (80). Conversely, right-hemispheric dominance and, especially, bilateral representation of language function in the WT should be interpreted with caution. In these situations, an additional assessment of the language lateralization by fMRI would be recommended, plus extra or intraoperative ESM for localization of language areas if an extensive temporal or frontal resection is planned.

### Functional MRI compared to the Wada test for language lateralization

**Figure 2:**
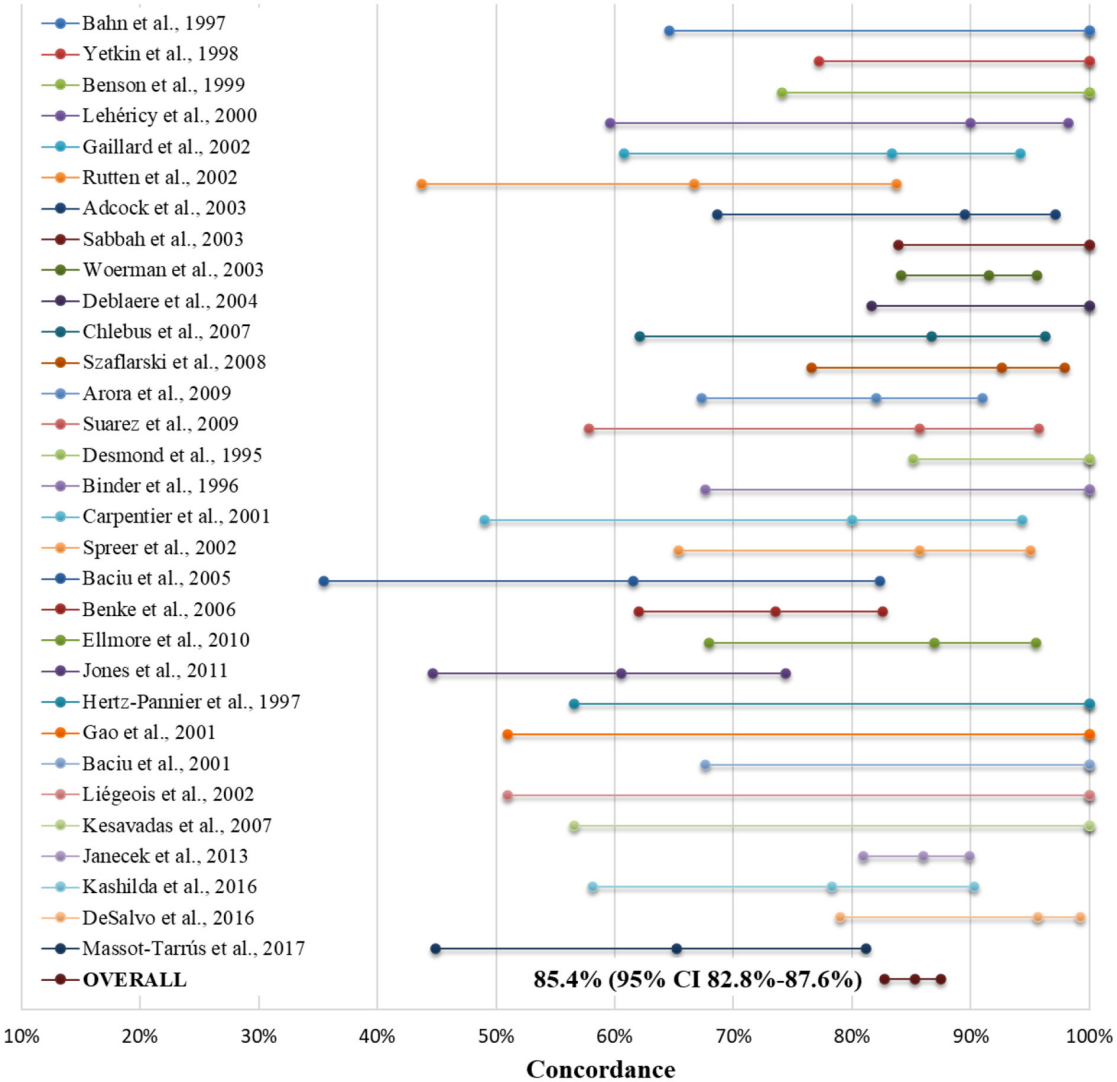
Forest plot indicating concordance between the Wada test and fMRI for language lateralization (as mean estimates and confidence bars) in each of the 31 studies included in our meta-analysis, both separately and as the sum of all patients (as "Overall"). For further details, see Massot-Tarrús et al., (5). Adopted with minor modifications from the original figure published in Massot-Tarrús et al. (5).

Taking the WT as the gold standard, numerous studies have compared fMRI and WT concordance for language lateralization. Most of them reported an excellent concordance between both tests using fMRI verb generation or semantic decision tasks (64, 84–89). In any case, the concordance was higher in right than left TLE (64, 90) and for frontal than TL activation (91). Furthermore, it was observed that to identify bilateral language dominance, it was important to use combined frontal/expressive and temporal/receptive language tasks (92). Even several meta-analyses have been performed comparing WT and fMRI for language lateralization in TLE. The largest and most recent (2017) was designed by our group and included 30 studies plus a series of 23 cases from our own Epilepsy Unit, for a total of 848 patients (5). The mean number of patients per study was 27.4, with a wide range—from 4 (93) to 229 (94)—and the vast majority (77.2%) of the cases had typical left-hemispheric lateralization for language in the WT. The concordance with fMRI and WT was a remarkable 85.4% on average (95% CI 82.8–87.6%), but with a wide variability (from 60.5% to a high of 100%). For further details, see Figure 2.

It is worth mentioning that a previous meta-analysis comprising 442 cases from 23 series (51) found a higher specificity of fMRI compared to WT with fMRI use of word generation compared to semantic decision tasks (95.6 vs. 69.5%) and in right-handed compared to ambidextrous or left-handed individuals (51). They attributed the latter to the higher rate of atypical language lateralization in the non-right-handed population (51). Supporting this, Bauer et al. observed that the concordance between WT and fMRI was lower in cases with atypical (bilateral or right) language lateralization (51 vs. 94%, respectively) analyzing 22 studies. One-third of the mismatched patients had typical left-hemispheric dominance in the WT but bilateral representation in fMRI (8). Likewise, studies in pediatric patients, a population with a well-known higher rate of bilateral or atypical language representation, have shown a lower concordance between both tests (95). The WT has falsely classified atypical language dominance as typical according to ESM and surgical outcomes in previous reports where fMRI classification was correct (96–98). Therefore, it is more likely that the discordant cases are correctly classified as having atypical language lateralization by fMRI and falsely classified as having typical language lateralization by the WT. These findings indicate that fMRI has a higher sensitivity for bilateral language representation than WT, which tends to overview this feature (8).

Finally, the WT-fMRI concordance was higher in mTLE compared to extratemporal epilepsies (87 vs. 81%, respectively) in another meta-analysis performed by the American Academy of Neurology, including exclusively class I and II studies (78).

In conclusion, fMRI can determine all the areas that are involved, although not necessarily essential, for language functions. In contrast, the WT only determines the hemisphere where the main functions are using object naming tasks. For these reasons, fMRI is more likely to detect bilateral representations for language functions or crossed dominance (expressive and receptive language functions in different hemispheres), whereas the WT is more likely to falsely classify atypical language dominance as typical (5, 96). A combination of fMRI paradigms that activate both the frontal/expressive language regions (e.g., verb generations, antonym generations, or object naming tasks) and temporal/receptive language areas (e.g., sentence completion and passive story listening tasks) would be recommended for this purpose (5, 68).

### Reliability and prognostic value of the fMRI for language after TLE surgery

The value of preoperative fMRI maps to predict the postoperative decline in language function has been fairly consistent (29, 52, 57, 82, 99). Specifically, Rosazza et al. (52) found that preoperative language performance accounted for 68% of the variance in postoperative language outcomes, and fMRI language lateralization explained an additional 16%. Stronger left-hemispheric activation during verbal fluency, verb generation, semantic decision, or auditory or picture naming tasks predicted naming decline 12 months after a left ATL with good sensitivity (52, 57, 82). Naming tasks primarily activate the part of the TL that is to be removed in ATL and seem to be more specific to predict naming difficulties than verbal fluency tasks—which mostly activate the inferior frontal gyrus (52, 57). Precisely, stronger fMRI activation of the left posterior inferior temporal area and fusiform gyrus during auditory and picture naming tasks have a consistent association with greater naming decline after left TL surgery, with picture naming being even more specific (100, 101). Right-sided lateralization of picture naming activations also appears to correlate with a greater postsurgical naming decline in right TLE (100). In another study, half of picture naming decline after TL surgery was explained by damage to a cluster of voxels in the fusiform gyrus approximately 4 to 6 cm posterior to the temporal tip activated by semantic decision tasks (102). Furthermore, You et al. reported that the most significant contributor to the naming decline was the resection of the top 10% of fMRI-activated regions during a word-definition task. Hence, such highly activated areas should be preserved from resection (99).

Few series have compared the ability of fMRI and the WT to predict postoperative naming decline (5). Sabsevitz et al. (82) examined 24 patients with left TLE using an fMRI semantic decision task. They found that language lateralization toward the resected left TL by both fMRI and the WT exhibited 100% sensitivity to predict naming decline, although fMRI had higher specificity (57 vs. 43%) (82). In the same line, Janecek et al. (103) analyzed 10 patients with left mTLE and discordant fMRI and the WT results. They found that fMRI was more reliable at predicting post-ATL naming outcomes than WT. Furthermore, as mentioned earlier, it has not been demonstrated that patients with bilateral language representation in the WT have a lower risk for language decline (64, 83), and fMRI seems more accurate at predicting lower risk due to right-hemispheric dominance (100). Therefore, fMRI seems more suited to elucidate language outcomes after TLE surgery than WT. Finally, patients with structural lesions or epileptogenic zone involving an indispensable language region (such as the Wernicke's area) from an early age are more likely to have a displacement of its functions to contiguous or contralateral regions (28). A bilateral or contralateral fMRI activation of the affected language region would support this displacement, which should be confirmed with intra- or extra-operative ESM.

### Measures to reduce the postoperative language decline in TLE

Resection of dispensable temporal language areas—such as the anterior part of the superior temporal gyrus, basal temporal region, and the long segment of the arcuate fasciculus in the dominant hemisphere—is typically not a reason to defer surgery because the visual naming impairment that it may produce usually recovers, at least partially, after several months (32, 33). At the same time, using ESM to identify and avoid the inclusion of the basal temporal area of language within the limits of ATL may help to further prevent postoperative naming decline (104, 105). For this reason, in the case of exploring the dominant TLE with stereo-electroencephalography (sEEG), implantation of deep electrodes targeting the anterior and posterior ventral TL cortex for language mapping would be recommendable (15).

Selective amygdala-hippocampectomy (SAH) is a more restricted surgical procedure that largely preserves the cortical areas. However, reduced language comprehension and fluency have been observed after SAH on the dominant side as well. This may be due to the deafferentation in cortical areas, disruption of basal temporal language area pathways, or neocortical lesions secondary to the different surgical approaches (106). Precisely, the transsylvian SAH approach transects the temporal stem and may also damage the superior temporal gyrus; the transcortical access transects the middle or inferior temporal gyrus (107, 108); and the subtemporal approach transects the parahippocampal and fusiform gyrus; thus damaging the temporo-basal naming areas. Initial studies reported that the transsylvian approach caused worse phonemic fluency than transcortical SAH (107, 109), and noncomparative series have suggested worse verbal learning, naming, and fluency with subtemporal SAH (107–110). Regarding this, Park et al. described a technical modification of the SAH subtemporal approach *via* the parahippocampal gyrus which preserves the fusiform gyrus, temporal stem, and the remaining temporal gyri. Such a technique should favor a better postsurgical language outcome, although this assumption has yet to be confirmed (111).

MRI-guided stereotactic laser ablation (SLA) technology also allows highly tailored resection, with minimal collateral damage to functional tissue. All available data suggest that SAH with SLA better preserves functions dependent on extra-mesial TL structures such as category-related naming, verbal fluency, and object and people recognition compared to open ATL or transcortical SAH (112, 113). On the other hand, the efficacy for seizure control appears to be slightly lower with SLA than with open resection techniques, especially in non-lesional surgeries (113–115).

The likelihood of long-term postoperative language deficit seems to be correlated with the distance between the resection margin and the indispensable cortical sites determined by ESM. However, no such correlation has been found with the distance from the language areas determined by fMRI (116). Resections have generally been tolerated up to 1 cm from these essential language sites, although some reports suggest that equivalent rates of permanent deficits occur when resections are performed without leaving a margin from positive ESM positive sites (41, 117). It is important to note, however, that this “no-margin technique” does have higher rates of transient postoperative deficits (118). A sufficient safety distance is presumed in standard ATL where the posterior extent of resection is limited to 4.5 cm from the temporal tip in the dominant TL (vs. 5.5 cm in the nondominant TL) (119).

When indispensable areas for language functions located within the epileptogenic zone aimed to be resected, the surgical options are limited. However, it should be taken into account that the traditional Broca's and Wernicke areas are no longer considered “unresectable” but rather nodes within a wide circuit, capable of self-reorganization, thanks to dynamical plasticity, especially in the presence of structural lesions or epileptic foci. It has been demonstrated in low-grade glioma awake surgery that the anterior language area can be safely resected provided that a small portion of the cortex located anterior to the left ventral premotor cortex and contiguous to subcortical language tracts is spared. The same holds true for the angular gyrus and posterior part of the superior temporal gyrus in the posterior language area (18, 120). The dynamical plasticity allows treatment strategies based on multistep surgical approaches too. These can include an evaluation by fMRI followed by intraoperative ESM before the surgical resection (18). In this regard, functional MRI and ESM may correspond but do not tend to overlap, with 60% to 75% of fMRI language signals lying within 1 cm of ESM demonstrated language areas (which can be up to 5 mm in the frontal language area) (29, 68, 121, 122). The concordance between both techniques is better with extra-operative than intra-operative ESM (123) and with the combination of language tasks using both auditory and visual input. Functional MRI can complement ESM for predicting postoperative language decline since higher sensitivity and specificity are obtained in the brain regions where both techniques overlap (124, 125). On the other hand, Rutten et al. (126) observed in 13 patients with TLE that only in 51% of cases, the temporoparietal language area in the fMRI was confirmed by intraoperative ESM. In any case, the absence of fMRI activity reliably predicted the absence of critical language areas on ESM (123, 126). So, fMRI has poor positive predictive value in determining indispensable areas for the language but can be used as an adjunct for preoperative planning (123).

Finally, multiple subpial transection is a palliative surgical technique that has been utilized to preserve cognitive functions, although long-term seizure control with this technique has been disappointing when performed on eloquent neocortical regions (127). Responsive neurostimulation is another treatment that does not result in cognitive decline and may even modestly improve the functions affected by the epileptic foci, like naming in patients with neocortical TLE (128).

### Indications for WT and fMRI use in clinical scenarios

Presurgical assessment for language is indicated in cases of potential atypical hemispheric dominance (right or bilateral) or cortical reorganization for language areas. These include left-handed or ambidextrous patients and left or bilateral TLE—-especially in case of epilepsy onset before 5 years of age or lesions involving the Wernicke area (19, 54). In other cases (i.e., right TLE in a left-handed individual), it would not be necessary before a standard ATL resection that should limit the posterior extent of resection to 4.5 cm in the left hemisphere (34, 119).

Functional MRI is the recommended test to evaluate language outcomes. However, the WT is still required in cases with inconclusive fMRI results, bilateral naming or language comprehension representation on fMRI, or strongly opposing lateralizing results by other methods used to determine language dominance (e.g., MEG, dichotic fused word listening test, etc.) (5). The reliability of fMRI is also less clear in neocortical TLE or lesional cases involving traditional language areas, especially when vascular malformations are involved (129). In such cases, the WT (130, 131) may also be indicated.

If the language dominance cannot ultimately be predicted with confidence or is considered bilateral by both fMRI and WT, the hemisphere to be resected should be treated as the dominant hemisphere and traditional language areas avoided (132, 133). In the latter case, fMRI can guide the extra or intraoperative ESM (123), with higher sensitivity and specificity for language areas where the findings of both techniques coincide (134). It should be taken into account that fMRI detects all areas that are involved in a given function, but not necessarily indispensable regions. Therefore, while preoperative fMRI can serve as an adjunct, it cannot yet replace cortical and subcortical ESM which stands as the gold standard to individually delineate eloquent cortex and fascicles in the anterior and posterior language areas (15, 118).

In case of discrepancy between the fMRI and WT, it should be taken into account the higher reliability of the fMRI to detect bilateral or right hemisphere dominance, and it would be also recommendable to confirm the results with extra- or intra-operative ESM.

Another scenario in which the WT may still be needed is in patients unable to cooperate or tolerate fMRI (metallic implants, claustrophobic, mentally challenged, children, etc.). This requires a minimal degree of collaboration, however, to confirm the appearance of aphasia even with simplified protocols (74). MEG, PET, or other tests less sensitive to movement distortions—such as the dichotic fused word listening test, functional near-infrared spectroscopy, or functional transcranial Doppler—are alternatives to consider in these cases (94, 135).

**Figure 3:**
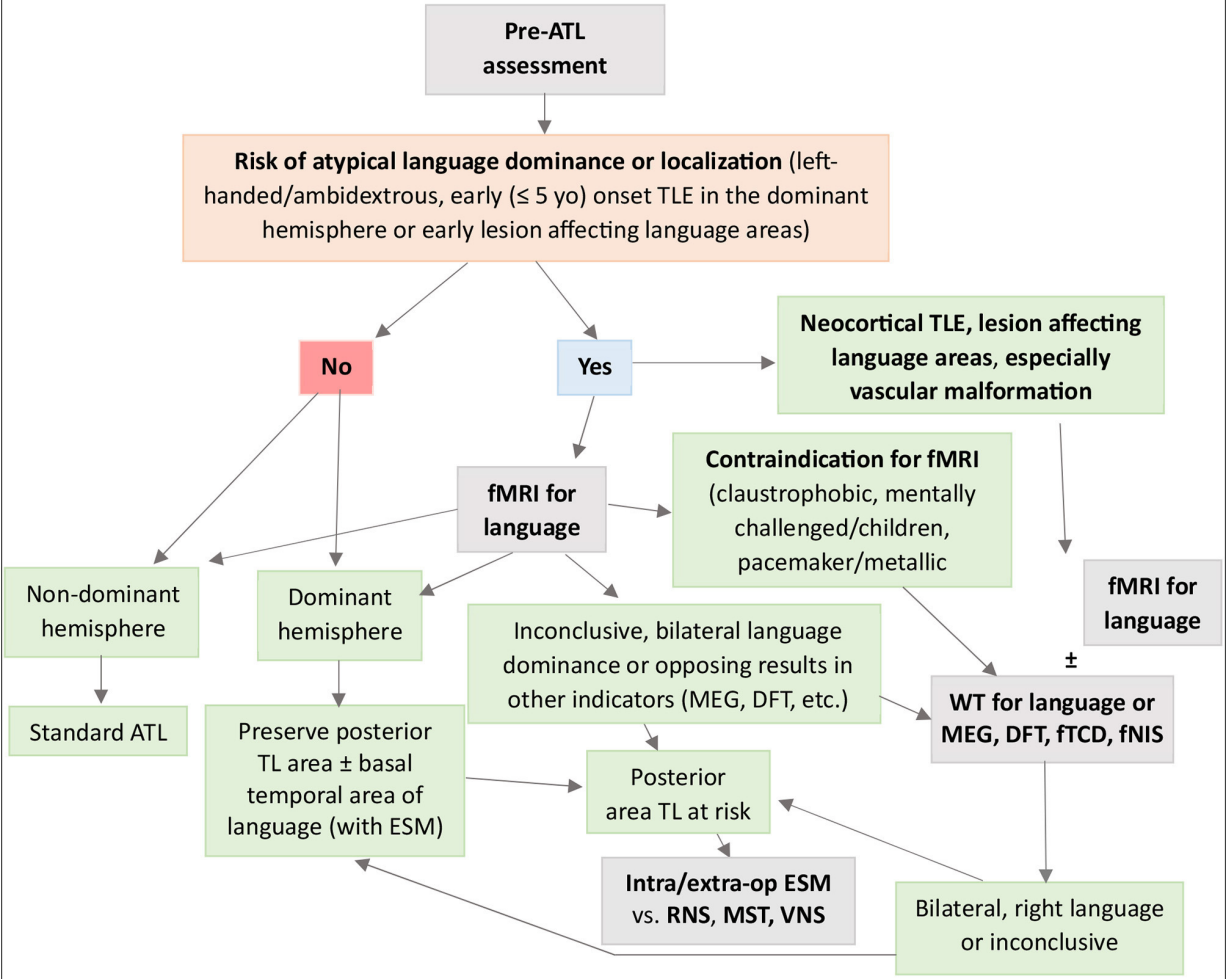
Flowchart to guide clinical decision-making when using fMRI and WT for presurgical evaluation of language in TLE. ATL, anterior temporal lobectomy; DFT, dichotic fused word listening test; fMRI, functional magnetic resonance imaging; fNIS, functional near-infrared spectroscopy; fTCD, functional transcranial Doppler sonography; Intra/extra-op ESM, intra/extra-operative electrocortical stimulation mapping; MEG, magnetoencephalography; MST, multiple subpial transections; RNS, responsive neurostimulation; TLE, temporal lobe epilepsy; VNS, vagus nerve stimulation; WT, Wada test.

A diagnostic algorithm for language evaluation in TLE is presented in Figure 3.

### Resting-state FMRI studies for language assessments

Resting-state fMRI determines brain functional connectivity (FC) in the absence of a task. Several networks have been identified in resting-state studies, and they include motor, visual, frontal, and default modes (136). Of particular interest is the default mode network (DMN), as it shows increased activation at rest compared to the activation seen during task performance. The DMN functionally links the posterior cingulate cortex, precuneus, mesial frontal region, inferior parietal area, and hippocampus. It has been linked to various cognitive processes (137) and consistently shows altered connectivity in multiple types of epilepsy (138, 139). Widespread decreases in FC, more significant in the hemisphere from which the seizures originate, have been identified in patients with TLE and can occur early in the disease process (136).

Regarding language function, Roger et al. (140) found that increased FC between the left hippocampus, left inferior frontal gyrus, and the supplementary motor area within the language-memory network was related to better naming, phonological, and semantic fluency, respectively. Also, Zhang et al. (141) observed that decreased FC between the left frontoparietal network and the visual network was associated with poor naming ability. DeSalvo et al. (142) identified high concordance between rs-fMRI—using independent component analysis (ICA) to calculate the language LI—and the WT for language (96% accuracy). However, the results were less consistent for patients with atypical language laterality. Similarly, Smitha et al. (143) obtained a high concordance of hemispheric language dominance between rs-fMRI and task-fMRI. Finally, Rolinski et al. (144) found moderate concordance between language LI from task fMRI and rs-fMRI in TLE using two novel methods for calculating resting-state language LI from Broca's area.

## Discussion and conclusion

Language functions of the brain are based on interconnected streams involving cortical and subcortical areas. Specifically, a left-lateralized white matter stream traveling dorsal to the Sylvian fissure engaged in sensory-motor mapping of sound to articulation; and a ventral stream, more bilaterally distributed responsible for mapping the auditory input onto conceptual and semantic representation (18). At the cortical level, there is a high degree of interindividual variability, which make necessary individualized approaches to each patient and brain region. The main regions involved in the semantic system are the anterior temporal pole (34), middle temporal gyrus (29, 35), inferior and latero-posterior temporal cortex (36, 145), and angular gyrus (37, 38, 40). The mid and posterior portions of the superior temporal gyrus are engaged in phonological analysis and naming production (29, 48).

Surgery on the TL of the dominant hemisphere for language implies a higher risk for language deficits. It is therefore mandatory to determine whether the TL to be operated is the dominant one. This is particularly the case in left-handed or ambidextrous patients and left or bilateral TLE—especially with onset before 5 years of age or with lesions or the epileptic zone involving the Wernicke area (19, 54, 100, 103). This is because early lesions and early onset of TLE promote brain plasticity and facilitate the reorganization of language tracts and areas to contiguous regions or the contralateral hemisphere (40, 41). At the same time, this adaptative neuroplasticity, together with the paradigmatic shift from localizationism to connectomics, implies that surgery can be performed in brain areas that were previously considered inoperable, like Wernicke's (18).

Functional MRI is the recommended test for the presurgical evaluation of language. A stronger activation during verbal fluency, verb generation, semantic decision, or auditory or picture naming to the ipsilateral hemisphere predicts naming decline after left and right ATL (52, 57, 82, 100). Naming tasks primarily activate the part of the TL that is to be removed in ATL and seem to be more specific to predicting naming difficulties (52, 57). Actually, the resection of the top 10% of fMRI-activated regions should be particularly avoided since it significantly contributes to naming decline (99).

Functional MRI shows a high concordance with the WT (5), which is still considered the gold standard for language lateralization. The WT determines the hemisphere where the key language structures lay and it is reliable to predict left-hemispheric dominance. However, it entails a significant risk of complications (65, 66), and its trustworthiness decreases when predicting right-hemispheric or bilateral language distribution (79–81). In this last respect, the fMRI lateralization with the combined use of expressive and receptive language tasks seems more reliable (92).

However, the WT is still necessary in some clinical scenarios: (a) when fMRI is inconclusive or shows bilateral language representations in any domain (naming, comprehension, etc.); (b) when fMRI shows opposing results to other studies to determine language dominance (e.g., MEG, dichotic fused word listening test, etc.); (c) in the patient unable to cooperate or tolerate fMRI; (d) and possibly, in neocortical TLE or lesional cases involving language areas, because the fMRI could be less precise in these situations.

Selective amygdala-hippocampectomy, especially with the subtemporal approach *via* the parahippocampal gyrus, could favor a better postsurgical language outcome in mTLE (111). Even less collateral cortical and subcortical damage and better functional outcome are obtained with SLA, which is indicated preferably in lesional mTLE (113–115).

If a resection near indispensable language areas—the posterior part of the inferior frontal gyrus, posterior part of the superior temporal gyrus, and angular gyrus (15, 31)—is planned, a minimum safety distance of 1 cm determined by ESM would be recommended, although equivalent rates of permanent deficits have been reported with “no-margin” techniques (41, 117). Functional MRI can complement ESM planification (124, 125) and a multistep surgical approach can be adopted (18). In this regard, fMRI has shown a poor positive but good negative predictive value to identify language regions (123, 126). Such security distance is presumed in standard ATL where the posterior extent of resection should be limited to 4.5 cm in the dominant TL (119).

Finally, rs-fMRI is a task-free technique with great potential that can detect altered connectivity in language networks in TLE. It may help to predict language outcomes in patients with limited cooperation due to disability or altered consciousness (e.g., coma or anesthesia) (146, 147). However, its specificity for determining language dominance requires further evaluation (44).

## Data availability statement

The original contributions presented in the study are included in the article/supplementary material, further inquiries can be directed to the corresponding author.

## Author contributions

AM-T designed the manuscript, conducted the bibliographic research, designed and wrote the manuscript, and generated Figure 3. SM contributed to the conceptualization and revised the manuscript. All authors contributed to the article and approved the submitted version.

## Funding

This study was funded by the London Health Sciences Foundation.

## Conflict of interest

AM-T has received honoraria for speaking and advisory board engagements from Bial, Eisai, UCB Pharma, and GW pharmaceuticals, as well as research support from Eisai and UCB. He has been involved in multiple multicenter clinical trials unrelated to fMRI and WT. SM is on the advisory boards and speaker bureaus for UCB Canada Inc., Eisai Limited, and Sunovion Pharmaceuticals Canada, Inc. He has been involved in multiple multicenter clinical trials unrelated to fMRI and WT.

## Publisher's note

All claims expressed in this article are solely those of the authors and do not necessarily represent those of their affiliated organizations, or those of the publisher, the editors and the reviewers. Any product that may be evaluated in this article, or claim that may be made by its manufacturer, is not guaranteed or endorsed by the publisher.
