# Erratum: Roles of fMRI and Wada tests in the presurgical evaluation of language functions in temporal lobe epilepsy

**DOI:** 10.3389/fneur.2023.1342365

**Published:** 2023-12-12

**Authors:** Frontiers Production Office

**Affiliations:** Frontiers Media SA, Lausanne, Switzerland

**Keywords:** intracarotid amobarbital test, functional MRI, resting-state, epilepsy surgery, naming, outcome

Due to a production error, some arrows in Figure 3 shifted and were incorrect in the published version. The correct figure appears below.

**Figure 3:**
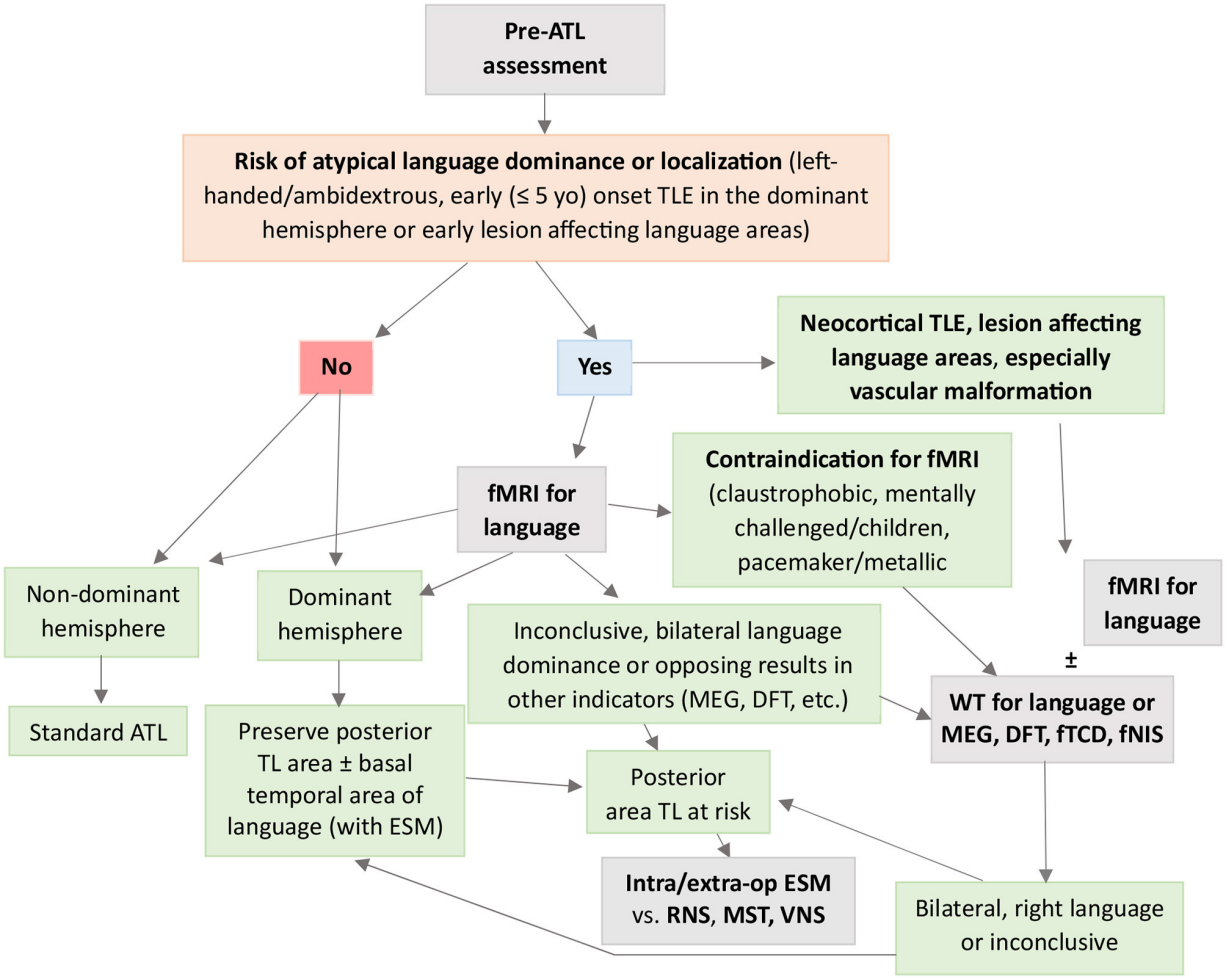
Flowchart to guide clinical decision-making when using fMRI and WT for presurgical evaluation of language in TLE. ATL, anterior temporal lobectomy; DFT, dichotic fused word listening test; fMRI, functional magnetic resonance imaging; fNIS, functional near-infrared spectroscopy; fTCD, functional transcranial Doppler sonography; Intra/extra-op ESM, intra/extra-operative electrocortical stimulation mapping; MEG, magnetoencephalography; MST, multiple subpial transections; RNS, responsive neurostimulation; TLE, temporal lobe epilepsy; VNS, vagus nerve stimulation; WT, Wada test.

The publisher apologizes for this mistake. The original article has been updated.

